# Supplementary material for: Differential Expression of Genes Involved in Metabolism and Immune Response in Diffuse and Intestinal Gastric Cancers, a Pilot Ptudy
Source: Biomedicines. 2022 Jan 23;10(2):240. doi: 10.3390/biomedicines10020240 (PMC8869420; doi:10.3390/biomedicines10020240)
Supplement: Supplementary file 1 [file biomedicines-10-00240-s001.zip › biomedicines-1531683-supplementary.pdf]

Supplementary Materials:

Table S1: mRNA expression of selected genes in intestinal- and diffuse- GC relative to non-tumoral tissue. [a]p value (Mann-Whitney test) ; significant p value<0.05 in bold ; % of under and over-expression.

Genes	non-tumoral gastric tissue (n=11)	intestinal GC (n=16)	p-value[a]	% under expression	% normal expression	% over expression	diffuse GC (n=13)	p-value[a]	% under expression	% normal expression	% over expression
immuno											
**PD receptor**	1 (0.35-2.80)	1.71 (0.81-5.30)	**0.003**	0%	81%	19%	1.53 (0.87-3.08)	**0.009**	0%	92%	8%
**PDL1**	1 (0.57-2.70)	1.46 (0.52-6.63)	0.08 (NS)	0%	69%	31%	1.03 (0.57-1.56)	0.84 (NS)	0%	100%	0
**PDL2**	1 (0.54-1.63)	1.67 (0.70-2.79)	**0.014**	0%	100%	0	1.21 (0.89-2.84)	**0.037**	0%	0%	0
**Tryptophan metablolism**											
**IDO1**	1 (0.19-1.46)	3 (0.34-205)	**0.0006**	0%	50%	**50%**	1.96 (0.57-4.8)	**0.002**	0%	77%	23%
**TDO2**	1 (0.45-2.95)	7.45 (1.4-25.2)	**<0.0001**	0%	20%	**80%**	3.33 (1.36-11.9)	**0.0004**	0%	46%	**54%**
**Aryl hydrocarbon receptor**											
**AhR**	1 (0.37-1.64)	1.60 (0.65-3.53)	0.001	0%	88%	12%	2.12 (0.55-3.35)	<0.001	0%	92%	8%

Table S2. Correlation of selected genes analysed in the study in relation to immunity and tryptophan metabolism in all GCs. r, Spearman 'rank test (relation between two parameters). Values in bold are statistically significant at confidence level greater than 99% (p value<0.01 and r>0.5).

Genes	PD1 r	PD1 P value	PDL-1 r	PDL-1 P value	PDL2 r	PDL2 P value	IDO1 r	IDO1 P value	TDO2 r	TDO2 P value
**immunity**										
**PD1**	x	x	**0,667**	**< 0.0001**	**0,641**	**0.0002**	0,473	**0,01**	0,352	0,06
**CD274/PDL1**	**0,667**	**< 0.0001**	x	x	0,310	0,10	**0,650**	**0.0001**	0,498	**0,006**
**PDL2**	**0,641**	**0.0002**	0,310	0,10	x	x	0,158	0,41	**0,529**	**0.003**
**IDO1**	0,473	**0,01**	**0,650**	**0.0001**	0,158	0,41	x	x	0,411	**0,03**
**TDO2**	0,352	0,06	0,498	**0,006**	**0,529**	**0.003**	*0,411*	**0,03**	x	x
**AHR**	0,115	0,55	0,162	0,40	0,146	0,45	0,287	0,13	0,051	0,79
**EMT and migration**										
**VIM**	0,068	0,72	-0,365	0,05	**0,519**	**0.004**	**-0,561**	**0.002**	-0,039	0,84
**CDH1**	0,430	**0,02**	0,494	0.006	0,066	0,74	0,318	0,09	0,221	0,25

**SNAI1**	0,296	0,12	0,249	0,19	0,397	**0,03**	0,130	0,50	0,500	**0.006**
**TGFB1**	**0,517**	**0.003**	0,356	0,06	**0,733**	**< 0.0001**	0,192	0,32	0,433	**0,02**
**cell prolif and migration**										
**Ki67**	0,180	0,35	**0,536**	**0.003**	-0,150	0,45	**0,614**	**0.0004**	0,357	0,06
**MMP2**	0,110	0,57	-0,332	0,08	**0,532**	**0.003**	-0,460	**0,01**	0,014	0,94
**MMP9**	**0,590**	**0.001**	**0,839**	**< 0.0001**	0,404	**0,03**	**0,526**	**0.003**	**0,676**	**< 0.0001**
**CXCR4**	**0,550**	**0.002**	0,210	0,27	0,367	0,05	0,142	0,46	0,070	0,70

Table S3: Correlation of selected genes analysed in the study in relation to immunity and tryptophan metabolism in intestinal GCs. r, Spearman ʻrank test. Values in bold are statistically significant.

**Genes**	**PD1**<br>**r**	**PD1**<br>P value	**PDL-1**<br>**r**	**PDL-1**<br>P value	**PDL2**<br>**r**	**PDL2**<br>P value	**IDO1**<br>**r**	**IDO1**<br>P value	**TDO2**<br>**r**	**TDO2**<br>P value
**immunity**										
**PD1**	x	x	**0,738**	**0.001**	0,482	0,06	0,451	0,08	0,194	0,47
**CD274/PDL1**	**0,738**	**0.001**	x	x	0,138	0,61	**0,680**	**0.004**	0,321	0,23
**PDL2**	0,482	0,06	0,162	0,61	x	x	0,119	0,94	0,450	0,08
**IDO1**	0,450	0,08	**0,680**	**0.004**	0,020	0,94	x	x	0,121	0,65
**TDO2**	0,194	0,48	0,320	0,23	0,450	0,08	0,121	0,65	x	x
**AHR**		0,56		0,45	-0,010	0,95	0,271	0,31	0,038	0,86
**EMT and migration**										
**VIM**	0,051	0,86	-0,303	0,25	**0,674**	**0.004**	-0,515	0,54	0,135	0,62
**CDH1**	0,583	0,02	**-0,600**	**0.01**	0,047	0,86	0,279	0,29	-0,032	0,90
**SNAI1**	0,295	0,25	0,141	0,60	**0,624**	**0.01**	-0,156	0,56	0,556	0,03
**TGFB1**	0,436	0,09	0,209	0,44	**0,760**	**< 0.001**	0,044	0,87	0,338	0,20
**cell prolif and migration**										
**Ki67**	0,135	0,61	0,523	0,04	-0,193	0,47	**0,686**	**0.002**	0,115	0,67
**MMP2**	0,082	0,76	-0,373	0,15	0,586	**0.02**	-0,538	0,03	-0,050	0,85
**MMP9**	0,517	0,04	**0,656**	**0.01**	0,473	0,06	0,238	0,37	0,571	0,02
**CXCR4**	**0,773**	**0.0004**	0,524	0,04	0,421	0,10	0,541	0,03	0,338	0,20

Table S4: Correlation of selected genes analysed in the study in relation to immunity and tryptophan metabolism in diffuse GCs. r, Spearman 'rank test. Values in bold are statistically significant.

**Genes**	**PD1** **r**	**PD1** P value	**PDL-1** **r**	**PDL-1** P value	**PDL2** **r**	**PDL2** P value	**IDO1** **r**	**IDO1** P value	**TDO2** **r**	**TDO2** P value
**immunity**										
**PD1**	x	x	0,479	0,10	**0,753**	**0.003**	0,437	0,13	0,377	0,20
**CD274/PDL1**	0,479	0,10	x	x	0,382	0,19	**0,630**	**0.02**	0,480	0,10
**PDL2**	**0,753**	**0.003**	0,382	0,19	x	x	0,374	0,21	0,500	0,08
**IDO1**	0,437	0,13	**0,630**	**0.02**	0,374	0,21	x	x	**0,604**	**0.03**
**TDO2**	0,377	0,20	0,480	0,10	0,506	0,08	**0,604**	**0.03**	x	x
**AHR**	0,530	0,07	0,419	0,17	0,327	0,27	0,530	0,07	0,253	0,43
**EMT and migration**										
**VIM**	0,344	0,25	-0,247	0,36	0,564	**0.04**	-0,297	0,32	0,154	0,62
**CDH1**	0,113	0,71	-0,302	0,31	0,027	0,93	0,209	0,49	0,121	0,69
**SNAI1**	0,165	0,59	0,044	0,89	-0,953	0,52	0,247	0,41	0,555	0,05
**TGFB1**	**0,610**	**0.03**	0,577	**0.04**	**0,638**	**0.02**	0,522	0,07	**0,621**	**0.03**
**cell prolif and migration**										
**Ki67**	0,058	0,85	0,077	0,80	-0,184	0,55	0,495	0,09	0,396	0,18
**MMP2**	0,355	0,23	-0,253	0,41	0,465	0,11	-0,136	0,68	0,467	0,11
**MMP9**	0,470	0,10	0,632	**0,02**	0,531	0,06	**0,709**	**< 0.01**	**0,737**	**0.005**
**CXCR4**	0,582	0.04	0,779	**0.0004**	**0,615**	**0.03**	**0,665**	**0.01**	0,225	0.46
